# Low-grade epilepsy-associated neuroepithelial tumors: Tumor spectrum and diagnosis based on genetic alterations

**DOI:** 10.3389/fnins.2022.1071314

**Published:** 2023-01-09

**Authors:** Mingguo Xie, Xiongfei Wang, Zejun Duan, Guoming Luan

**Affiliations:** ^1^Department of Neurosurgery, Epilepsy Center, Sanbo Brain Hospital, Capital Medical University, Beijing, China; ^2^Beijing Key Laboratory of Epilepsy, Sanbo Brain Hospital, Capital Medical University, Beijing, China; ^3^Department of Pathology, Sanbo Brain Hospital, Capital Medical University, Beijing, China; ^4^Beijing Institute for Brain Disorders, Capital Medical University, Beijing, China; ^5^Chinese Institute for Brain Research, Beijing, China

**Keywords:** brain tumor, neuroepithelial, diagnosis, pathology, epilepsy

## Abstract

Brain tumors can always result in seizures when involving the cortical neurons or their circuits, and they were found to be one of the most common etiologies of intractable focal seizures. The low-grade epilepsy-associated neuroepithelial tumors (LEAT), as a special group of brain tumors associated with seizures, share common clinicopathological features, such as seizure onsets at a young age, a predilection for involving the temporal lobe, and an almost benign course, including a rather slow growth pattern and thus a long-term history of seizures. Ganglioglioma (GG) and dysembryoplastic neuroepithelial tumor (DNET) are the typical representatives of LEATs. Surgical treatments with complete resection of tumors and related epileptogenic zones are deemed the optimal way to achieve postoperative seizure control and lifetime recurrence-free survival in patients with LEATs. Although the term LEAT was originally introduced in 2003, debates on the tumor spectrum and the diagnosis or classification of LEAT entities are still confusing among epileptologists and neuropathologists. In this review, we would further discuss these questions, especially based on the updated classification of central nervous system tumors in the WHO fifth edition and the latest molecular genetic findings of tumor entities in LEAT entities.

## Introduction

Actually, every brain tumor involving the neocortex or neuronal circuits thereof can result in seizures (Stone et al., 2018b). Brain tumors have been found to be the second most common histopathological diagnosis among the surgical specimens from patients with epilepsy, second to focal cortical dysplasia (FCD) in children and hippocampal sclerosis (HS) in adults (Blumcke et al., 2017). Some brain tumors, however, grow rather slowly and are specifically prone to occurring in young patients and primarily presenting with seizures (Luyken et al., 2003; Blumcke et al., 2017). The term “long-term epilepsy-associated tumors (LEATs)” was thus originally introduced by Luyken et al. (2003), when recognizing that tumors were more commonly encountered in surgical series of patients who had been treated for drug-resistant epilepsy with such long-term seizure onsets as more than 2 years. Notably, ganglioglioma (GG) and dysembryoplastic neuroepithelial tumor (DNET) are the classical representatives of this category of tumors (Englot et al., 2012; Giulioni et al., 2017). Since then, more and more cases of brain tumors with epilepsy have been reported, and the concept of LEATs has been gradually recognized (Thom et al., 2012; Slegers and Blumcke, 2020). LEATs are the collective name of a group of tumors with different histological features in each entity (Luyken et al., 2003; Thom et al., 2012; Slegers and Blumcke, 2020). Despite the large morphological variability in LEATs, commonalities were also reported as follows: (1) seizure onsets begin at a young age (usually 12–15 years), without significant sex preference (Luyken et al., 2003; Wessling et al., 2015; Blumcke et al., 2017; Giulioni et al., 2017; Faramand et al., 2018); (2) tumors occur with preference of the temporal involvement (approximately 65–80%) of either left or right brain hemisphere (Giulioni et al., 2017; Ristić et al., 2020; Slegers and Blumcke, 2020); and (3) the majority of LEAT entities are mixed glioneuronal tumors (GNT), belonging to benign neoplasms and assigned to WHO grade 1, with rather slow growth patterns and very few cases of malignant progression, and thus accompanied by a long-term seizure history (usually > 2 years) (Luyken et al., 2003; Wessling et al., 2015; Ehrstedt et al., 2017; Pelliccia et al., 2017). Surgical treatments with complete resection of tumors and associated epileptogenic zones (EZ) are recognized as the optimal approach to achieve postoperative seizure control and lifetime recurrence-free survival for patients with LEATs (Luyken et al., 2003; Englot et al., 2012; Blumcke et al., 2017; Shan et al., 2018).

Although relevant in clinical practice, several aspects of the concept of LEATs have been questioned. First of all, the term was originally applied to brain tumors associated with long-term (>2 years) drug-resistant epilepsy (Luyken et al., 2003), but the definition of refractory epilepsy has become less strict since the term was proposed (Wessling et al., 2015; Radhakrishnan et al., 2016; Stone et al., 2018a; Ko et al., 2019). Particularly in children with epilepsy, the strategy of early neuroimaging screening and surgical intervention, if possible, has been encouraged to prevent abnormal brain development and future neurocognitive deficits caused by recurrent seizures (Blümcke et al., 2016; Pelliccia et al., 2017; Vogt et al., 2018). Thus, changing the phrase “long-term” in LEATs to “low-grade” has been proposed, as the majority of LEAT entities are truly low-grade neoplasms (Blümcke et al., 2016; Slegers and Blumcke, 2020). Recently, the term “low-grade developmental epilepsy-associated brain tumors” was also introduced among researchers in recognition of the fact that most LEAT entities belong to developmental glioneuronal tumors, such as GG and DNET, which are rather related to the occurrence of FCD (Palmini et al., 2013; Aronica and Crino, 2014). More specifically, as Blümcke et al. proposed, the definition of LEATs was changed to “low-grade epilepsy-associated neuroepithelial tumors” to indicate such distinguishable pathological features of LEATs as low-grade and neuroepithelial from other groups of tumors with epilepsy (Blumcke et al., 2014; Blümcke et al., 2016; Slegers and Blumcke, 2020). However, this term does not fit into the WHO concept of nosology in tumor classification, which is based on specific cell types, for instance, astrocytoma, pineocytoma, meningioma, etc. (Louis et al., 2016, 2021). In addition, debates on, in particular, the tumor spectrum and the diagnosis or classification of LEAT entities are still controversial and always confusing among epileptologists and neuropathologists (Thom et al., 2012; Blümcke et al., 2016; Slegers and Blumcke, 2020). In the review, we also quoted the nosology of “low-grade epilepsy-associated neuroepithelial tumors,” with an abbreviation of LEATs, and we would like to further discuss these debatable aspects of LEATs mentioned above.

### The spectrum of brain tumors in LEAT

Since the terminology of LEATs was proposed, a large number of brain tumors with neuroepithelial origination have been included in the tumor spectrum of LEATs (Thom et al., 2012; Phi and Kim, 2019; Slegers and Blumcke, 2020). The tumor spectrum of established LEAT entities is broad and has significantly increased according to the fourth WHO classification update (Table 1; Blümcke et al., 2016; Slegers and Blumcke, 2020; Louis et al., 2021). However, except for the established tumors of GG and DNET, other tumors in LEATs are not yet well-recognized due to their rather low incidences, especially from a single center report with limited cases (Blümcke et al., 2016; Blumcke et al., 2017), and thus they are variably reported in the surgical series of LEATs (Luyken et al., 2003; Wessling et al., 2015; Radhakrishnan et al., 2016; Giulioni et al., 2017; Vogt et al., 2018; Ristić et al., 2020), including angiocentric glioma (AG) (Ni et al., 2015; Wang et al., 2020), papillary glioneuronal tumor (PGNT) (Bridge et al., 2013; Pages et al., 2015; Hou et al., 2019), multinodular and vacuolating neuronal tumor (MVNT) (Gonzalez-Quarante et al., 2018; Pekmezci et al., 2018a; Thom et al., 2018; Choi et al., 2019; Gökçe, 2020), isomorphic astrocytoma/isomorphic diffuse glioma (IDG) (Wefers et al., 2020; Appay et al., 2021), pilocytic astrocytoma (PA) (Blümcke et al., 2016), and sometimes including pleomorphic xanthoastrocytoma (PXA) (Weber et al., 2007), diffuse low-grade gliomas (DLGGs) of diffuse astrocytoma (DA) and oligodendroglioma (d-OT) or oligoastrocytoma (d-OA) (Luyken et al., 2003; Vogt et al., 2018; Ius et al., 2020), and the newly diagnosed entity of “polymorphous low-grade neuroepithelial tumor of the young (PLNTY)” (Huse et al., 2017; Louis et al., 2021). Although shared clinical features are found in these lesions, arguments still exist in the categorization of which tumor entities are true LEATs (Blümcke et al., 2016; Slegers and Blumcke, 2020). Reviewing the case reports in the literature, the less common tumor entities of AG, PGNT, MVNT, and PA, plus the classical representatives of GG and DNET, are gradually regarded as the traditional members of the LEAT family (Ko et al., 2019; Ristić et al., 2020). However, debates could be found on the remaining entities of PXA, IDG, PLNTY, and even low-grade DA and d-OT/OA, with inconsistent results of the tumor spectrum in the LEAT group from different surgical series (Thom et al., 2012; Phi and Kim, 2019; Slegers and Blumcke, 2020).

**TABLE 1:** The grouping of low-grade gliomas, glioneuronal/neuronal tumors based on the 2021 WHO classification of CNS tumors and the tumor spectrum of LEAT.

Gliomas, glioneuronal tumors, and neuronal tumors	Abbreviation	WHO grading	Traditional LEAT entities	Characteristic genes/Molecular profiles
**Part 1. Diffuse glioma**				
**1. Adult-type diffuse gliomas**				
Astrocytoma, IDH-mutant	DA	2/3/4^†^	N	IDH1, IDH2, ATRX, TP53, and CDKN2A/B
Oligodendroglioma, IDH-mutant, and 1p/19q-codeleted	d-OT	2/3^†^	N	IDH1, IDH2, 1p/19q, TERT promoter, CIC, FUBP1, and NOTCH1
**2. Pediatric-type diffuse gliomas**				
**2.1 Pediatric-type diffuse low-grade gliomas**				
Diffuse astrocytoma, MYB- or MYBL1-altered	p-DA/(IDG)*	1	Y	MYB and MYBL1
Angiocentric glioma	AG	1	Y	MYB
Polymorphous low-grade neuroepithelial tumor of the young	PLNTY	1	Y	BRAF and FGFR family
Diffuse low-grade glioma, MAPK pathway-altered	DLGG*	nd	nd	FGFR1 and BRAF
**Part 2. Circumscribed astrocytic gliomas**				
Pilocytic astrocytoma	PA	1	Y	KIAA1549-BRAF, BRAF, and NF1
High-grade astrocytoma with piloid features	HGAP	nd	N	BRAF, NF1, ATRX, and CDKN2A/B (methylome)
Pleomorphic xanthoastrocytoma	PXA	2/3^†^	N	BRAF and CDKN2A/B
Subependymal giant cell astrocytoma	SGCA	1	N	TSC1 and TSC2
Chordoid glioma	CG	2	N	PRKCA
Astroblastoma, MN1-altered	AB	nd	N	MN1
**Part 3. Glioneuronal and neuronal tumors**				
Ganglioglioma	GG	1/3^†^	Y	BRAF
Desmoplastic infantile ganglioglioma/astrocytoma	DIG/DIA	1	N	nd
Dysembryoplastic neuroepithelial tumor	DNET	1	Y	FGFR1
Diffuse glioneuronal tumor with oligodendroglioma-like features and nuclear clusters	DGONC	nd	nd	Chromosome 14 (methylome)
Papillary glioneuronal tumor	PGNT	1	Y	PRKCA
Rosette-forming glioneuronal tumor	RGNT	1	N	FGFR1, PIK3CA, and NF1
Myxoid glioneuronal tumor	MGNT	nd	N	PDFGRA
Diffuse leptomeningeal glioneuronal tumor	DLGNT	nd	N	KIAA1549-BRAF fusion, 1p (methylome)
Gangliocytoma	GC	1	N	BRAF
Multinodular and vacuolating neuronal tumor	MVNT	1	Y	MAPK pathway
Dysplastic cerebellar gangliocytoma (Lhermitte-Duclos disease)	DCG (LDD)	1	N	PTEN
Central neurocytoma	CN	2	N	nd
Extraventricular neurocytoma	EVN	2	N	FGFR (FGFR1-TACC1 fusion), IDH-wild-type
Cerebellar liponeurocytoma	CLN	2	N	nd

LEAT, low-grade epilepsy-associated neuroepithelial tumors; nd, not defined; Y, yes; N, no.

*IDG, isomorphic diffuse glioma with MYB or MYBL1 alterations, equally to the new tumor type of “diffuse astrocytoma, MYB- or MYBL1-altered” in Pediatric-type (p-DA) group classified by the 2021 WHO classification; DLGG, diffuse low-grade glioma, MAPK pathway-altered, which as a new tumor type was not defined by WHO panel with specific tumor grading.

^†^The high WHO grades of 3/4 indicate tumor subtype with anaplasia or malignancy in the new 2021 classification.

Pleomorphic xanthoastrocytomas are an astrocytic tumor that predominantly occurs in children and young adults and usually has a relatively favorable behavior when compared to diffuse glial tumors in adults (Louis et al., 2016; Vaubel et al., 2021). PXAs account for less than 1% of all astrocytic tumors and have a typical superficial meningocerebral location, often with the involvement of the temporal lobe, in nearly 70–80% of cases (Luyken et al., 2003; Blumcke et al., 2017; Giulioni et al., 2017). PXAs are semi-benign brain tumors that share molecular and morphological commonalities with traditional LEATs, such as CD34 immunoreactivity in 73% of cases of PXAs (Thom et al., 2012) and BRAF^V600E^ mutation in 50–75% of analyzed PXAs (Schindler et al., 2011). Recently, a homozygous deletion of CDKN2A/B, corresponding to the loss of 9q21.3, was found as a rather distinctive molecular feature of PXA, regardless of tumor grade or BRAF mutation (Vaubel et al., 2018). Patients with PXAs often present with seizures and are thus frequently represented in epilepsy surgery series within the spectrum of LEATs, accounting for 2% of all brain tumors in epilepsy surgery (Blumcke et al., 2017; Qi et al., 2018). However, some authors did not treat PXA as a true LEAT entity, due to their semi-malignant nature and WHO tumor grading of grade 2 and grade 3 with anaplasia (Slegers and Blumcke, 2020; Vaubel et al., 2021). PXAs are always found with relatively high tumor recurrence and malignant transformation than other entities in LEATs, with 5-year progression-free and overall survival of 59.9–70.9 and 80.8–90.4%, respectively, in grade 2 cases, and with more aggressive behavior and decreased 5-year overall survival of 47.6–57.1% in tumor with anaplasia (Ida et al., 2015; Vaubel et al., 2018, 2021).

Diffuse low-grade gliomas usually refer to DA and d-OT in previous case reports, regardless of age grouping (Phi and Kim, 2019). These tumors are commonly found developing in young adults and involve large areas of the brain cortex and subcortical areas, most notably the frontal lobes (Roberts et al., 2018; Ius et al., 2020). Seizure onsets are the most common manifestation of DLGGs, and nearly 80–90% of patients with DLGGs had seizures (Pallud and McKhann, 2019; Ius et al., 2020). Frequently, however, DLGGs have been excluded from the discussion of epilepsy-associated tumors because the majority of DLGGs correspond to histopathological WHO grade 2 tumors with a far higher rate of infiltration, recurrence, and malignant progression than typical LEATs (Blümcke et al., 2016; Ko et al., 2019; Slegers and Blumcke, 2020). DLGGs are thus considered a true invasive neoplasm that should be dealt with in the oncology field (Duffau, 2018). However, many patients with DLGGs attain long-term survival and subsequently face the same problem of long-standing seizures as patients with LEATs. In fact, many surgical cohorts of epilepsy-associated tumors have included a number of patients with DLGGs in addition to the backbone of the traditional LEAT entities, especially when adolescents or young adults are included (Luyken et al., 2003; Wessling et al., 2015; Radhakrishnan et al., 2016; Vogt et al., 2018). The differences in clinicopathological features between DLGG in adults and children have been highlighted in a variety of surgical series (Luyken et al., 2003; Phi and Kim, 2019; Ius et al., 2020). Particularly, according to the 2021 WHO classification of central nervous system (CNS) tumors, the DLGG have been divided into adult and pediatric types (Louis et al., 2021), and the adult-type DLGG (Astrocytoma, IDH-mutant; Oligodendroglioma, IDH-mutant, and 1p/19q-codeleted) are recognized as truly invasive neoplasms with a higher risk of tumor progression and malignant transformation (Duffau and Taillandier, 2015; Jones et al., 2018; Lombardi et al., 2020). Furthermore, these tumors are more likely to present with symptoms of increased intracranial pressure and/or focal neurological deficits, or with a shorter history of seizures, and thus should be differently treated from tumor entities of LEATs (Phi and Kim, 2019; Lombardi et al., 2020; Young et al., 2020).

In contrast, the pediatric-type DLGG, which includes four tumor types, namely, DA (MYB/MYBL1-altered), AG, PLNTY, and DLGGs (MAPK pathway-altered), is considered benign tumors and assigned as WHO grade 1, and they have been found to be more related to the LEATs (Table 1; Slegers and Blumcke, 2020; Louis et al., 2021). For example, the PLNTY was described by Huse et al. (2017) in 2017 as a distinct epileptogenic neoplasm within the spectrum of pediatric, low-grade neuroepithelial tumors. This group of tumors presented in 10 patients with infiltrative growth patterns, a predominant oligodendroglioma-like glial cell component, and intense CD34+ as the most common features. All 10 patients were diagnosed at a young age, with a mean age of 17 years (4–32 years old), with 8/10 seizures, and with 7/10 temporal locations that are similar to LEAT entities (Huse et al., 2017). Molecular analysis revealed a BRAF^V600E^ mutation, FGFR2 fusion, and FGFR3 fusion in 3/8, 3/8, and 1/8 tested tumors, respectively. This kind of tumor is recognized by the WHO panel of CNS tumor classification as a new tumor type, mainly because they represent a high proportion of low-grade oligodendroglial tumors in children and should be distinguished from other low-grade tumors with a distinct DNA analysis (Huse et al., 2017; Riva et al., 2018; Louis et al., 2021).

In addition, the pediatric-type DAs with MYB/MYBL1 alterations have also been reported to be closely related to the LEATs that are quite different from DAs with IDH mutations in adults (Bergthold et al., 2014; Vogt et al., 2018; Ius et al., 2020). For instance, previous clinical neuropathological studies have found that postoperative tumor progression and recurrence are less often in patients with DAs with a long history of seizures than those with a very short history of seizures (Luyken et al., 2003; Schramm et al., 2004), indicating a tumor subtype presenting with better prognosis in patients with chronic epilepsy, the so-called “isomorphic astrocytoma” (Blümcke et al., 2004), which recently was renamed by Wefers et al. (2020) as “isomorphic diffuse glioma (IDG),” a group of tumors clearly distinct from other glial/glioneuronal brain tumors (Louis et al., 2021). These astrocytoma variants are characterized by a supratentorial, highly differentiated glioma with low cellularity, low proliferation, and focal diffuse brain infiltration. Patients typically had seizures since childhood and were operated on as adults, with excellent progression-free survival after resection (Blümcke et al., 2004; Thom et al., 2012). Interestingly, 77% of IDGs demonstrated MYM/MYBL1 alterations, and all (100%) were IDH-wild-type, which are closely related to pediatric MYB/MYBL1-altered diffuse astrocytomas, according to the WHO fifth edition of CNS tumor classification (Wefers et al., 2020; Louis et al., 2021). Thus, these pediatric-type MYB/MYBL1-altered DAs or IDGs probably represent a distinct group of genetically defined LEATs (Blümcke et al., 2016; Slegers and Blumcke, 2020).

In fact, based on the more biologically and molecularly defined entities of CNS tumors, the 2021 WHO fifth edition classification separated the low-grade neuroepithelial tumors from those with higher infiltration or WHO grading. Furthermore, most of the cortex-involved tumors in the subgroups of “Pediatric-type diffuse low-grade gliomas,” “Circumscribed astrocytic gliomas” and “Glioneuronal and neuronal tumors” are regarded as benign entities with a rather slow growth pattern and thus can result in a long-term history of epilepsy that would much relate to the LEATs (Table 1; Blümcke et al., 2016; Slegers and Blumcke, 2020; Louis et al., 2021). Indeed, several tumor types have been reported in different surgical cohorts of epilepsy-associated neuroepithelial tumors, such as AG and PLNTY in the subgroup of “Pediatric-type diffuse low-grade gliomas” (Bandopadhayay et al., 2016; Huse et al., 2017; Han et al., 2020), PA and PXA in the subgroup of “Circumscribed astrocytic gliomas” (Wallace et al., 2011; Jones et al., 2013; Collins et al., 2015) and GG, DNET, PGNT, and MVNT in the subgroup of “Glioneuronal and neuronal tumors” (Blümcke et al., 2016; Slegers and Blumcke, 2020; Louis et al., 2021). Herein, we propose that the LEAT entities could be roughly grouped into the three subgroups mentioned above, and any new tumor types found in these subgroups in the future could be potential members of the LEAT family and thus be treated by epilepsy surgery in the neurosurgery department. However, for these tumors, further classification requires precise molecular analyses, notably based on the integration of histopathological and molecular information in a tiered diagnostic format as Louis et al. (2021) had recommended recently. Future studies, especially between multiple epilepsy therapeutic centers, are required to improve and standardize the terminology of LEATs and to extend the use of molecular genetic diagnostic tools over a histomorphology-based classification to specify clinically meaningful tumor entities that could be included in the LEAT spectrum.

### Molecular genetic alterations and diagnoses in LEAT

Although the tumor spectrum of LEATs has been widely discussed since nosology was introduced, the histopathological diagnosis and classification of tumors in LEATs remain challenging due to their variable histopathological features (Qaddoumi et al., 2016; Stone et al., 2018a; Slegers and Blumcke, 2020), which include varieties of cellular components, such as astroglia, oligodendroglia, neoplastic or pre-existing neurons, and inflammatory cellular infiltrates, as well as multiple architectural growth patterns, including nodular or cyst growth and even diffuse infiltration of tumor cell clusters at sites distant from the tumor mass with or without calcification (Blümcke and Wiestler, 2002; Thom et al., 2011). In addition, many glioneuronal tumors lack specific histological features that are crucial for the diagnosis of GG or DNET or have mixed histological features in the same specimen. For example, 5–20% of case series have mixed GG and DNET or PXA histological components (Prayson and Napekoski, 2012; Qaddoumi et al., 2016; Faramand et al., 2018; Stone et al., 2018a). Furthermore, LEAT-associated FCD, namely FCD IIIb (Blumcke et al., 2011), is another complex issue in need of clarification, with highly variable proportions of 10–75% (Prayson, 2011; Giulioni et al., 2017; Pelliccia et al., 2017).

To make a more accurate diagnosis or classification of the LEATs, many ways have been tried to assist in tumor diagnosis by purely microscopic inspection of pathological tissue, especially for some tumors with limited tissue specimens from piece-meal resection or by biopsy (Thom et al., 2012; Blümcke et al., 2016). Immunohistochemistry with staining for CD34, P16, S100, MAP2, GFAP, NeuN, and synaptophysin is helpful, but these markers are not so specific (Thom et al., 2012). Recently, combined molecular pathological diagnosis is widely discussed in the LEAT group (Table 2; Qaddoumi et al., 2016; Stone et al., 2018a). Simultaneously, the current 2021 WHO classification of CNS tumors has also recommended some specific molecular genetic signatures for the neuropathological diagnosis of low-grade neuroepithelial tumors (Table 1; Louis et al., 2016). However, the genetic biomarkers that have been unraveled for LEATs have not yet been systematically reviewed in a large and consecutive cohort of LEATs due to their low incidences. Meanwhile, parts of the molecular genetic signatures are shared by more than one tumor type (Table 2). This dilemma finally contributes to the long-lasting challenge of achieving a reliable differential diagnosis of tumors in the LEAT group (Horbinski et al., 2011; Blümcke et al., 2016). Thus, as Louis et al. (2014, 2021) have recommended, it requires the integration of histopathological and molecular information in a tiered diagnostic format for precisely differentiating the diagnosis and classification of tumors, also in the LEAT group. Herein, we exclusively conclude the recent findings that are helpful to make a more accurate diagnosis or classification of LEATs, including the histological and molecular genetic aspects of each entity.

**TABLE 2:** The molecular genetic alterations in each tumor subtype of LEAT summarized from different case reports in the literature*.

Genetic alterations	BRAF^V600E^ mutations (%)	FGFR1/(2/3) alterations (%)	MYB/MYBL1 (MYB-QKI fusion) (%)	SLC44A1-PRKCA fusion (%)	Other genetic alterations
GG	18.2–57.7% (Sievert et al., 2009; Dougherty et al., 2010; Schindler et al., 2011; Chappé et al., 2013; Dahiya et al., 2013; Koelsche et al., 2013; Ramkissoon et al., 2013; Zhang et al., 2013; Prabowo et al., 2014; Qaddoumi et al., 2016; Pekmezci et al., 2018b)	16% (Huse et al., 2017)	/	/	RAF1 (3%), KRAS (5%), NF1 (3%), FGFR1 (5%), FGFR2 (8%), ABL2 (3%), CDKN2A (8%), and PTEN (3%) (Pekmezci et al., 2018b)
DNET	29.8–51% (Chappé et al., 2013; Prabowo et al., 2014; Ross et al., 2014)	58.1–81.8% (Qaddoumi et al., 2016; Rivera et al., 2016)	/	/	/
AG	13.3% (Qaddoumi et al., 2016)	/	66–100% (Ramkissoon et al., 2013; Bandopadhayay et al., 2016; Qaddoumi et al., 2016)	/	MYB-ESR1 fusion, QKI rearrangement (Qaddoumi et al., 2016)
PGNT	/	/	/	39.3–100% (Pages et al., 2015; Hou et al., 2019)	NOTCH1-PRKCA fusion (Hou et al., 2019)
PXA	60.5–65.5% (Schindler et al., 2011; Ida et al., 2015; Vaubel et al., 2018)	/	/	/	CDKN2A/B (83 and 93%) (Weber et al., 2007)
MVNT	25% (BRAF not V600E) (Pekmezci et al., 2018a)	12.5–14.3% (FGFR2) (Pekmezci et al., 2018a; Choi et al., 2019)	/	/	MAP2K1,(Pekmezci et al., 2018a) DEPDC5, SMO, and TP53 (Thom et al., 2018)
IDG	/	/	77% (54%, MYBL1; 23%, MYB) (Wefers et al., 2020)	/	/
PLNTY	37.5% (Huse et al., 2017)	12.5–37.5% (12.5%, FGFR3; 37.5%, FGFR2) (Huse et al., 2017)	/	/	/
PA	9.3% (33%, extra-cerebellar) (Schindler et al., 2011)	/	/	/	NF1, KRAS, the NTRK family, and FGFR1 (Jones et al., 2013; Zhang et al., 2013)
DA	17–29% (Ramkissoon et al., 2013; Zhang et al., 2013; Cruz et al., 2014; Roth et al., 2014; Qaddoumi et al., 2016)	17% (Zhang et al., 2013)	26–41% (Ramkissoon et al., 2013; Zhang et al., 2013; Qaddoumi et al., 2016)	/	/
d-OT	8% (Zhang et al., 2013)	40–69% (Zhang et al., 2013; Qaddoumi et al., 2016)	8% (Zhang et al., 2013)	/	/

AG, angiocentric glioma; DA, diffuse astroglioma; DNET, dysembryoplastic neuroepithelial tumor; d-OT, diffuse oligodendroglioma; GG, ganglioglioma; IDG, isomorphic diffuse glioma; LEAT, low-grade epilepsy associated neuroepithelial tumors; MVNT, multinodular and vacuolated neuronal tumor; PA, pilocytic astrocytoma; PGNT, papillary glioneuronal tumor; PXA, pleomorphic xanthoastrocytoma; PLNTY, polymorphous low-grade neuroepithelial tumor of the young.

*The molecular genetic alterations with their incidences were found in each tumor entity of LEAT and DA/d-OT from different reports in the literature, and the DA and d-OT include tumors occurring both in pediatric and adult groups thus the real rates of genetic alteration might be compromised in previous reports.

#### BRAF^V600E^ mutations in GG

The GG is a well-differentiated, slowly growing neuroepithelial tumor, with its biphasic composition of glial and neuronal cell elements first introduced by Perkins OC in 1926 (Wolf et al., 1994; Blümcke et al., 2016). GGs are the most common epilepsy-associated neoplasms that account for 50–60% of brain tumors in epileptic patients but only 1–2% of all primary brain tumors, and they are recognized by the WHO as a grade 1 tumor or a grade 3 tumor with anaplasia (Louis et al., 2016; Blumcke et al., 2017).

The BRAF^V600E^ mutation was found to be significantly related to GG, but different rates of BRAF^V600E^ mutation were reported from previous series of GG in surgical specimens, ranging from 18 to 56% (Sievert et al., 2009; Dougherty et al., 2010; Schindler et al., 2011; Chappé et al., 2013; Dahiya et al., 2013; Koelsche et al., 2013; Ramkissoon et al., 2013; Zhang et al., 2013; Prabowo et al., 2014; Pekmezci et al., 2018b). Interestingly, Koh et al. (2018) further confirmed the pathogenic role of the BRAF^V600E^ mutation in an animal model that BRAF^V600E^ induced epileptogenesis in the neuronal lineage and tumorigenesis in the glial lineage. Since the first BRAF^V600E^-specific antibody was reported in 2011 (clone VE1) (Capper et al., 2011), it has been widely used nowadays to screen for BRAF^V600E^ mutations in the diagnostic work-up of tissue specimens. In particular, several clinicopathological features, such as seizure onset, tumor progression, and postoperative seizure outcome, have been investigated in relation to BRAF mutations. For example, Vornetti et al. (2017) found multiple seizure types were present in patients with LEATs and BRAF^V600E^ mutation but none with the BRAF^V600E^ wild type (*p* = 0.035); Dahiya et al. (2013) and Chen et al. (2017) found the worse recurrence-free survival was related to the BRAF^V600E^ mutation in GG cohorts. Furthermore, Prabowo et al. (2015) investigated a cohort of GNTs with BRAF^V600E^ mutations detected in 38/93 (40.8%) GGs and 23/77 (29.8%) DNETs by immunohistochemistry and found the expression of BRAF^V600E^ was associated with a worse postoperative seizure outcome in GNTs (*p* < 0.001). However, other case reports did not find any significant associations of BRAF mutations with patient age, seizure onset, tumor progression or recurrence, and seizure outcome (Shen et al., 2017; Vornetti et al., 2017; Pekmezci et al., 2018b; Stone et al., 2018a). Thus, further studies are required to investigate the possible role of BRAF^V600E^ mutations, such as being a prognostic marker of tumor behavior and seizure outcome, in epilepsy-associated tumors (Martinoni et al., 2015).

It is noteworthy that the BRAF^V600E^ mutation is not much specific to GG. As reported by Pekmezci et al., the BRAF^V600E^ mutation was screened in a cohort of 1320 nervous system tumors, and the mutation was found more frequently in PXA (66%) than WHO grade 1 GG (18%) and PA (9%) (Schindler et al., 2011). In addition, DNET (30–50%) (Chappé et al., 2013; Prabowo et al., 2014; Ross et al., 2014), AG (13%) (Qaddoumi et al., 2016), DA (17–29%) (Zhang et al., 2013; Cruz et al., 2014; Roth et al., 2014), and d-OT (8%) (Zhang et al., 2013) also share the BRAF^V600E^ alteration (Table 2). In addition, many other genetic alterations, but without IDH1/2, have also been described in GG, among which genetic alterations of the MAP kinase signaling pathway are most prominent (Horbinski et al., 2011). In a study of 40 GGs by Pekmezci et al. (2018b), for example, RAF1 (3%), KRAS (5%), NF1 (3%), FGFR1 (5%), FGFR2 (8%), ABL2 (3%), CDKN2A (8%), and PTEN (3%) were detected. Although the BRAF^V600E^ mutation could not be a such specific diagnostic marker in the genetic panel of brain tumors as GG, the differential diagnosis of GG can be established with the combination of its histological features with CD34 immunoreactive, BRAF^V600E^ mutation and IDH1/2 wild type (Blümcke et al., 2016; Slegers and Blumcke, 2020).

#### FGFR1 alterations in DNET

The DNET was originally described by Daumas-Duport et al. (1988), and it is histologically composed of a simple form with a unique glioneuronal element or a complex form with both glial nodules and glioneuronal elements, corresponding to WHO grade 1 (Blümcke et al., 2016; Louis et al., 2016). DNETs are the second most prevalent tumors associated with chronic or drug-resistant epilepsy and are frequently represented in the LEAT series, approximately 30–50% (Radhakrishnan et al., 2016; Faramand et al., 2018; Slegers and Blumcke, 2020).

FGFR1 gene alterations in DNET were first reported by Zhang et al. (2013). A more comprehensive study revealed FGFR1 alterations in 18 of 22 DNETs (82%), including 9 tyrosine kinase domain duplications, 8 missense single nucleotide variants, and 8 FGFR1-TACC fusions (Qaddoumi et al., 2016). Rivera et al. (2016) confirmed the above findings and showed 12 FGFR1 tyrosine kinase domain duplications, 10 point mutations, and 3 breakpoints in 25 of 43 DNETs (58%). However, FGFR1 alterations are also shared by other neuroepithelial tumors in various proportions, such as GG (16%) (Stone et al., 2018a), DA (17%) (Zhang et al., 2013), and d-OT (40–69%) (Zhang et al., 2013; Qaddoumi et al., 2016). In addition, BRAF^V600E^ alteration was also frequently documented in 30–51% of DNETs (Table 2), but without IDH1/2 mutation (Thom et al., 2011). Even so, the diagnosis of DNET, as with GG, can be established with the combination of its histological features with FGFR1 alterations, the BRAF^V600E^ mutation, and IDH1/2 wild type (Thom et al., 2011; Blümcke et al., 2016; Slegers and Blumcke, 2020).

#### MYB fusions in AG

The AG represents a rare, slowly growing cerebral glial tumor that has been recognized by the WHO as a grade 1 tumor (Blümcke et al., 2016). AGs often occur in children and young adults and are more frequently identified in the setting of chronic epilepsy, but only account for 0.5% of all epileptic patients with brain tumors (Blumcke et al., 2017; Han et al., 2020). AGs often involve the frontoparietal and temporal lobes and histopathologically are characterized by perivascular pseudorosettes with an ependymoma-like appearance (Bandopadhayay et al., 2016; Louis et al., 2016).

MYB fusions have been reported as rare events in pediatric low-grade gliomas and were first described in a total of 9 tumors of which two were AG (Zhang et al., 2013). This has been confirmed by Qaddoumi et al. (2016), who studied 15 AGs, and identified recurrent MYB alterations in all AGs assayed. Of the 15 cases analyzed, 13 (87%) possessed a MYB-QKI fusion, while the remaining 2 possessed a MYB-ESR1 fusion and a QKI rearrangement, respectively (Qaddoumi et al., 2016). The prevalence of MYB alterations in AG was repeated in a subsequent cohort of 19 tumors, all of which harbor MYB-QKI fusions (Bandopadhayay et al., 2016). This study also demonstrated that MYB-QKI fusion was able to drive tumorigenesis *via* simultaneous activation of MYB as a result of enhancer translocation combined with the loss of the tumor suppressor activity of QKI. Taken together, these data suggest that MYB abnormalities are sufficient as a specific and single-driver event in AG (Blümcke et al., 2016; Stone et al., 2018b). However, shared mutations of MYB/MYBL1 abnormalities can occur in other low-grade neuroepithelial tumors, including DA (26–41%) (Ramkissoon et al., 2013; Zhang et al., 2013; Qaddoumi et al., 2016), d-OT (8%) (Zhang et al., 2013), IDG (77%), MYBL1 (54%), MYB (23%) (Wefers et al., 2020), and DNET (in one case) (Zhang et al., 2013). In addition, Qaddoumi et al. (2016) found two tumors of AG with a MYB-QKI fusion also harbored a BRAF^V600E^ mutation (2/15) (Table 2).

#### PRKCA translocations in PGNT

The PGNT is a rare glioneuronal tumor first described in 1997 and was recognized in the WHO 2007 classification as an entity distinct from GG (Komori et al., 1998; Blümcke et al., 2016). PGNTs tend to be tumors of young adults with a mean age at presentation of 25.9 years (ranging from 4 to 75 years) (Hou et al., 2019; Slegers and Blumcke, 2020). A history of seizures was recorded in 30–50% of the reported PGNTs, and they approximately account for 0.1% of the epilepsy-associated brain tumors (Blumcke et al., 2017). PGNTs are composed of GFAP-positive astrocytes, lining hyalinized vascular pseudopapillae, SYN-positive, interpapillary collections of sheets of neurocytes, neurons, and “ganglioid” cells, attributed to WHO grade 1 (Thom et al., 2012; Pages et al., 2015; Blümcke et al., 2016).

Recently, a fusion of SLC44A1 and PRKCA, which encodes a protein kinase C involved in the MAP kinase signaling pathway, has been described in several studies (Bridge et al., 2013; Pages et al., 2015; Hou et al., 2019). Bridge et al. (2013) identified a recurrent chromosomal translocation *t*(9;17) (q31;q24), with a resultant oncogenic fusion protein SLC44A1-PRKCA, in three PGNTs. Pages et al. (2015) analyzed 4 pediatric PGNTs and 15 PGNT mimics. SLC44A1-PRKCA fusion occurred in all PGNTs, but none of the PGNT mimics, and all PGNTs were negative for BRAF and FGFR1 mutations. More recently, Hou et al. (2019) looked at 28 PGNTs using DNA methylation analysis and revealed that 11/28 of the tumors were true PGNT with a canonical SLC44A1-PRKCA fusion and the remainder of 17/28 tumors were other types of tumors due to previous incorrect histological classification, but an alteration of NOTCH1-PRKCA fusion was also found in PGNT (Table 2). These results reported in previous studies suggest that SLC44A1-PRKCA fusion can be a specific characteristic of PGNT with a high diagnostic value and be detectable by fluorescence *in situ* hybridization (FISH). Notwithstanding, further studies with molecular genetic information analyzed in a large case series of PGNT are still necessary to identify these genetic alterations.

#### Genetic alterations in MVNT

The MVNT was originally described by Huse et al. (2013) in 10 patients, which was subsequently confirmed by Bodi et al. (2014) in two additional patients. MVNTs are defined currently by the WHO as benign tumors (WHO grade 1) associated with seizures, predominately in the temporal lobe (Blumcke et al., 2017; Louis et al., 2021). These tumors are featured by clustering in multiple small nodules of vacuolating neuronal tumor cells and lacking cell proliferation and infiltration (Blümcke et al., 2016; Pekmezci et al., 2018a; Thom et al., 2018).

In a cohort of 7 MVNTs, no BRAF^V600E^ mutations were found, but one case showed a FGFR2 fusion (Choi et al., 2019). In another cohort of 8 MVNTs, genetic alterations were found in BRAF other than V600E, MAP2K1, and FGFR2 in 2/8, 5/8, and 1/8 of cases, respectively (Pekmezci et al., 2018a). Interestingly, all of these genetic alterations are converging on the activation of the MAP kinase signaling pathway and are found to be related to the tumorigenesis and the resultant epileptogenesis (Koh et al., 2018; Delev et al., 2020; Drosten and Barbacid, 2020). Particularly, all cases of MVNT in previous reports with the molecular analysis are absent in BRAF^V600E^ mutations (Table 2; Thom et al., 2018; Choi et al., 2019). In addition, in a recent cohort of 10 MVNT cases, no mutations in FGFR1 or MYB were identified (Thom et al., 2018). Thus, given the prevalence of mutations affecting BRAF^V600E^, FGFR1, and MYB in other entities of LEATs, the absence of these genetic alterations in MVNT may be helpful to differentiate these tumors (Stone et al., 2018b). However, due to the diverse and limited molecular findings reported in the literature, more studies are needed to further understand the molecular genetics and etiology of this rare neoplasm (Stone et al., 2018b; Slegers and Blumcke, 2020).

**FIGURE 1:**
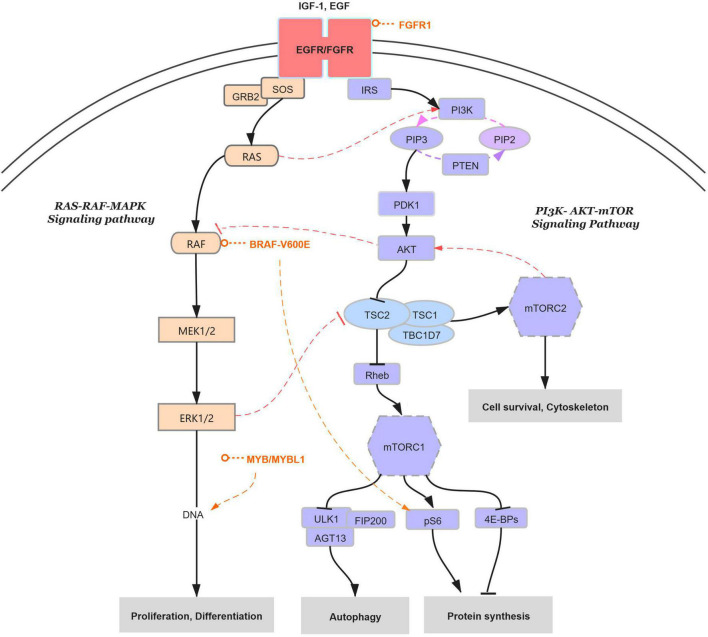
RAS-RAF-MAPK signaling pathway with molecular genetic alterations affected in LEATs. Genetic alterations detected in LEAT entities mainly involve two signaling cascades, namely, RAS-RAF-MAPK (left/pink) and PI3K-AKT-mTOR (right/blue). Signals begin at the insulin-like growth factor-1 (IGF-1) receptor at the cell surface, as well as the epidermal growth factor (EGF) receptor, and transmit to the downstream canonical cascades of the MAPK pathway (through RAS, RAF, and MEK1/2 to ERK1/2) and the mTOR pathway (through PI3K, PDK-1, AKT, and TSC1-TSC2-TBC1D7 complex to mTORC1/2). The specific genetic alterations are listed in the figure (light red), including the FGFR1 alteration and BRAF^V600E^ mutation detected in GG and DNET and the MYB/MYBL1 fusions found in AG and IDG, with the activation of the RAS-RAF-MAPK signaling pathway to control DNA transcriptions for cell proliferation and differentiation. In particular, the MAPK pathway activation is regulated by substrates of the PI3K-AKT-mTOR signaling cascade, which, in turn, was controlled by the components from RAS-RAF-MAPK cascades to determine the protein synthesis (dashed lines).

In summary, genetic alterations detected in LEAT entities involve and connect two major signaling pathways, namely, the mitogen-activated protein kinase (MAPK) pathway and the mammalian target of rapamycin (mTOR) pathway (Figure 1; Blümcke et al., 2016; Pernice et al., 2016; Delev et al., 2020). For example, FGFR1 as receptor signaling at upstream of both pathways has been identified in DNETs with FGFR1 alterations; BRAF as a substrate further downstream of the RAS-RAF-MAPK signaling cascade have been described in GG and DNET with BRAF^V600E^ mutations, which were always accompanied by the activation of mTOR signaling cascade with increased phosphorylated ribosomal S6 protein (pS6) (LaSarge and Danzer, 2014; Prabowo et al., 2014; Ehrstedt et al., 2020); in addition, c-MYB/MYBL1, as one of the regulated transcription factors of both signaling cascades, have also been demonstrated in IDG and AG with MYB-QKI fusion (Blümcke et al., 2016; Qaddoumi et al., 2016; Slegers and Blumcke, 2020). Particularly, the MAP kinase activation can be regulated by substrates of the PI3K-AKT-mTOR signaling cascade and vice versa, which have been identified as more related to focal malformations of cortical development (MCD), such as tuberous sclerosis complex (TSC), hemimegalencephaly, and FCD (Crino, 2015; Pernice et al., 2016). Interestingly, LEAT entities are also found to be closely related to the occurrence of MCD (Thom et al., 2012; Giulioni et al., 2017). In addition, molecular alterations of CD34 expression and BRAF mutation are often concurrently met in low-grade tumors, such as GG, DNT, and PXA. However, the relationships between CD34 expression and the BRAF mutation were still unknown in previous studies. Studies with molecular genetic information analyzed in a large case cohort of LEATs in the future are still required to further identify the genetic alterations and interactions of the two major signaling pathways (Blümcke et al., 2016), as well as the relationships between CD34 expression and the BRAF mutation.

### The epileptogenesis and surgical management of LEATs

Brain tumors result in 6–15% of seizure onsets in patients with epilepsy and 24–27% of focal seizures (Blumcke et al., 2017; Ertürk Çetin et al., 2017). Although our knowledge of molecular pathways driving neoplastic cell growth and malignant progression has gradually matured, the issues of why and how a seizure occurs in a patient with a brain tumor still need to be clarified (Slegers and Blumcke, 2020; Natale et al., 2021). Two main hypotheses have been proposed previously, namely, the tumor-centric and the epilepsy-centric approaches (van Breemen et al., 2007; Pallud et al., 2013). The tumor-centric approach states that the epileptic activity derives from the tumor itself, which was recently confirmed by the experimental work of Koh et al. (2018) in neurons transfected with the BRAF^V600E^ mutation *in vivo*. In addition, nearly half of patients would have seizure onsets completely controlled after the tumor resection alone (Englot et al., 2012; Bonney et al., 2015). The epilepsy-centric approach provides evidence that the infiltrated peritumoral neocortex is key for tumor-related epileptic activity, due to metabolic imbalances of glioma-related glutamatergic and γ-aminobutyric acid changes leading to epileptogenicity (Lee et al., 2007; Yuen et al., 2012; Pallud et al., 2013; Neal et al., 2016). In fact, many alterations have been found in human peritumoral brain tissue that has the potential to dramatically alter neuronal and glial homeostasis and the microenvironment and thus result in an epileptogenic state (Stone et al., 2018b; Maschio et al., 2019; Thomas and Pierson, 2020; Zhang et al., 2020).

These two epileptogenic hypotheses lead to another important issue of how to achieve complete seizure control after surgery (Stone et al., 2018b). However, LEATs were among the best candidates for complete postoperative seizure control, and approximately 75–90% of patients could get seizure-free after surgery (Luyken et al., 2003; Slegers and Blumcke, 2020). Planning for epilepsy surgery needs to take into consideration, therefore, any MRI-visible lesion as well as resecting of the ictal onset zone (Maschio et al., 2019). After all, a better seizure control was always documented in patients with the extensive resection of tumor and peritumoral EZ, which thus satisfies the surgical demands of both tumor-centric and epilepsy-centric approaches (Englot et al., 2012; Bonney et al., 2015; Shan et al., 2018).

## Discussion

The LEATs, as a distinct group of epilepsy-associated brain tumors, share common clinicopathological characteristics. Although the GG, DNET, AG, PGNT, MVNT, and PA are deemed the typical tumor entities in the LEAT spectrum, other new tumor entities, especially in the 2021 WHO edition of CNS tumors, are gradually being recognized with close association with LEATs, such as PLGTY and IDG (or pediatric-type diffuse astrocytoma with MYB/MYBL1 alteration), which, however, should be further identified in large cohorts. The LEAT entities always have a rather slow growth pattern, thus accompanying a long-term history of seizures, and complete seizure control with lifetime recurrence-free survival can be achieved after surgical resection. However, the histopathological heterogeneities of both morphological and cellular elements in LEAT entities always confuse neuropathologists, and thus the diagnosis of a specific neoplasm needs to combine the histomorphological features with the specific molecular genetic markers in each tumor, such as BRAF^V600E^, FGFR1, MYB, and PRKCA alterations. Notwithstanding, more collaborations, especially between multiple epilepsy therapeutic centers, should be underlined to improve and standardize the criteria and terminology of LEATs and to extend the use of molecular genetic diagnostic tools over a histomorphology-based classification to specify clinically meaningful tumor entities within the LEAT spectrum when considering the low incidence of these lesions. In addition, although several clinicopathological features, such as tumor progression and postoperative seizure outcome, have been reported related to molecular markers, especially, BRAF mutations, future studies are also needed to confirm these data in a larger, well-matched cohort of LEATs and to further investigate possible relationships between clinicopathological features and other molecular markers of LEAT entities as well.

## Author contributions

MX and GL wrote the manuscript. MX, XW, and ZD analyzed and interpreted the patient data regarding epilepsy-associated neuroepithelial tumors from the literature and our institute. All authors read and approved the final manuscript.
